# Enhancing Performance of Silicone Hydrogel Contact Lenses with Hydrophilic Polyphenolic Coatings

**DOI:** 10.3390/jfb15110321

**Published:** 2024-10-29

**Authors:** Paul Demian, Daichi Nagaya, Roeya Refaei, Kaoru Iwai, Daiki Hasegawa, Masaki Baba, Phillip B. Messersmith, Mouad Lamrani

**Affiliations:** 1 Menicon R&D Innovation Centre, Menicon Co., Ltd., Nagoya (Japan), Geneva Branch, 1205 Geneva, Switzerland; p.demian@meniconrd.com; 2 Menicon Co., Ltd., 21-19, Aoi 3, Naka-ku, Nagoya 460-0006, Japan; 3 Laboratory of LAMSE, Faculty of Sciences and Techniques of Tangier, Abdelmalek Essaâdi University, B.P. 416, Tangier 90000, Morocco; 4 Bioengineering and Materials Science and Engineering Departments, University of California, Berkeley, CA 94720, USA; philm@berkeley.edu

**Keywords:** dopamine, tannic acid, chitosan, lipid-repellent, silicone hydrogel

## Abstract

This study explores the application of a dopamine-assisted co-deposition strategy to modify the surface of daily disposable silicone hydrogel contact lenses. Aiming to enhance the hydrophilicity of these typically hydrophobic lenses, we developed an industry-friendly process using simple dip coating in aqueous solutions. By co-depositing tannic acid, dopamine and chitosan derivative and employing periodate oxidation, we achieved a rapid and efficient coating process. High-molecular-weight branched polyethylene imine was introduced to promote surface reactions. The resulting lenses exhibited extreme hydrophilicity and lipid repellency without compromising their intrinsic properties or causing cytotoxicity. While the coating demonstrated partial antimicrobial activity against Gram-positive Staphylococcus aureus, it offers a foundation for the further development of broad-spectrum antimicrobial coatings. This versatile and efficient process, capable of transforming hydrophobic contact lenses into hydrophilic ones in just 15 min, shows significant potential for improving comfort and performance in daily disposable contact lenses.

## 1. Introduction

Since first being reported by Messersmith et al. in 2007 [1], mussel-inspired chemistry has revolutionized surface modification strategies. Dopamine, which contains functional catechol and amino groups, can spontaneously oxidize and polymerize into polydopamine (PDA) under aerobic and alkaline conditions [2]. PDA coatings have emerged as a versatile tool for functionalizing nearly any material surface, including organic materials, metal oxides, and noble metals. The applications of PDA coatings are rapidly expanding into various fields including antimicrobial surfaces, tissue engineering and other branches of biomedical fields [3,4,5].

The intrinsic chemical reactivity of PDA, due to catechol-quinone moieties and catechol radical species, allows for spontaneous reactions with nucleophiles such as amines and thiolates, making it ideal for various post-modification strategies. However, post-functionalization chemicals are restricted to those containing amine or thiol groups, and the method suffers from a slow deposition rate [6]. In 2012 [7], Lee and Messersmith introduced a one-step dopamine-assisted co-deposition strategy by immersing various substrates into mixed solutions of dopamine and other chemicals, including polymerization initiators, antibacterial agents, tertiary amines and polysaccharides. This method allowed for the easy adjustment of surface properties by varying the content and molecular structure of the co-deposited components.

In this study, we explored the application of a dopamine-assisted co-deposition strategy in the contact lens industry. Specifically, we described a process to modify the surface of daily disposable silicone hydrogel lenses, which are typically hydrophobic due to the presence of siloxane polymers. Enhancing the hydrophilicity of these lenses is crucial for their efficacy as ophthalmic devices [8,9,10]. There are various approaches reported in the literature to achieve this modification [11,12,13]. Among these, polysaccharides such as chitosan and hyaluronic acid [14,15] have demonstrated high efficiency as hydrophilic surface agents or surface layers, significantly improving the wettability and performance of the lenses.

From an industrial standpoint, daily disposable lenses represent a product that is manufactured on a large scale to meet high customer demand. This high-scale production necessitates a short manufacturing time and results in a relatively low price per piece. Consequently, any technology integrated into this process must adhere to these criteria: efficiency in production time, cost-effectiveness and technological simplicity. By leveraging the adhesive and hydrophilic properties of PDA-based coatings, we improved the performance of daily disposable silicone hydrogel lenses, while ensuring that the manufacturing process remained efficient, simple and cost-effective.

In this paper, we aimed to communicate a set of specific design decisions that were determined experimentally to achieve a super-hydrophilic and industry-compatible coating for daily disposable silicone hydrogel contact lens materials. Firstly, we adopted a strategy of co-deposition of less colored and bulkier tannic acid with dopamine, which acts as a molecular anchor. Periodate oxidation was employed instead of changing the pH to dramatically reduce the reaction time. To promote the superficial reaction of co-deposition rather than aggregate formation in an aqueous solution, the very hydrophobic surface was pretreated with high-molecular-weight branched polyethylene imine. Furthermore, the hydrophilic properties of the resulting coatings were significantly enhanced by introducing a chitosan derivative as a co-deposition macromolecule. Once oxidized, the chitosan is transformed into dialdehyde chitosan [16], which acts as a macromolecular crosslinker [17] through Schiff base reactions between dopamine and tannic acid.

As a result, we developed a reliable chemical process capable of transforming a hydrophobic contact lens surface into a hydrophilic one in just 15 min. This process is extremely versatile and can be applied to any type of contact lens. The coating is durable and does not impact any of the product’s properties, making it highly suitable for industrial application.

## 2. Materials and Methods

### 2.1. Chemicals and Materials

All chemicals used in our protocols were purchased from Merk (Sigma-Aldrich Chemie GmbH), Buchs, Switzerland, except for Sodium meta-Periodate, which was purchased from Fischer Scientific AG, Reinach, Switzerland, and the chitosan derivative TmCs-MOI, synthesized on request by Nissui company in Tokyo, Japan.

**Figure 1:**
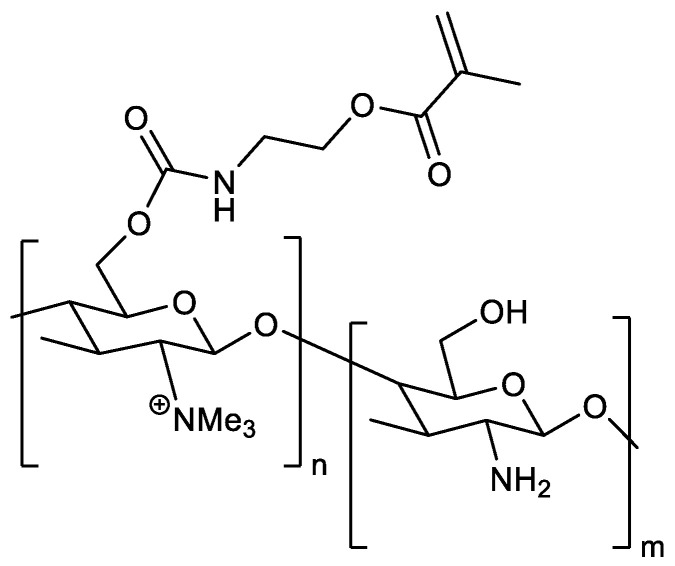
Chemical structure of Tm-Cs-MOI, a quaternized chitosan derivative with polymerizable acrylic function.

The characterization of TmCs-MOI (Figure 1) reported by the manufacturer revealed a molecular weight (Mn) of 38,381 with a polydispersity index (Mw/Mn) of 3.81. The degree of quaternization was determined to be 19.3%, and the degree of substitution of MOI was 17.0%. Additionally, the residual solvent content was found to be 0.03%.

### 2.2. Coating Solutions

Stock solutions were prepared as follows:

- Polyethylene imine (18.75 × 10^−3^ g/mL) was dissolved in deionized (DI) water.
- The chitosan derivative aqueous solution was prepared by dissolving TmCs-MOI (6.25 × 10^−3^ g/mL) in a buffer solution containing 0.1 M bicine and 0.6 M NaCl at pH 5.2.
- An oxidant solution was prepared by dissolving NaIO_4_ (12.5 × 10^−3^ g/mL) in DI water.
- A phenolic solution was prepared by dissolving dopamine chloride (3 × 10^−3^ g/mL) and tannic acid (15 × 10^−3^ g/mL) in the buffer solution containing 0.1 M bicine and 0.6 M NaCl at pH 5.2.

All solutions were sonicated for 5 min prior to their use.

### 2.3. Coating Process

**Figure 2:**
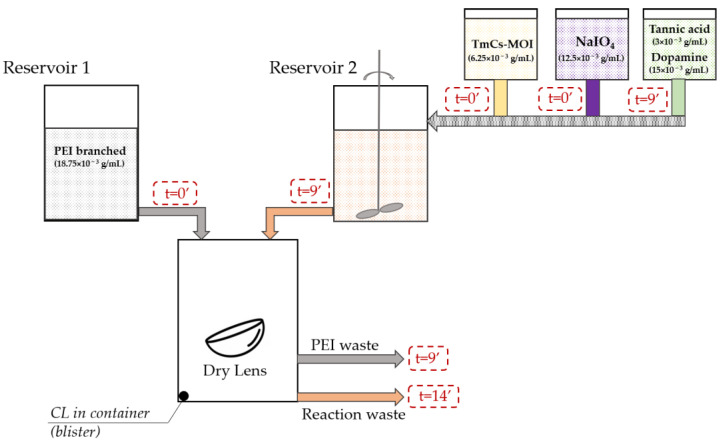
Schematic representation of the technological process of contact lens surface modification by dopamine-assisted co-deposition.

In the first stage, the contact lens (CL) container (or non-sealed blister) containing a lens in a dry state was filled with a polyethyleneimine (PEI) solution from Reservoir 1 (Figure 2). While the adsorption of PEI was ongoing, a second coating solution was prepared in a separate reservoir, Reservoir 2. Reservoir 2 was first filled with 2.4 mL of TmCs-MOI solution, followed by the addition of 0.3 mL oxidant solution. After 9 min of TmCs-MOI oxidation, 0.3 mL of the phenolic solution was added. Subsequently, the PEI solution from the lens container was replaced by the resulting solution of oxidized coating compounds and left to react for 5 min. The treated lens was then rinsed and autoclaved in the corresponding saline solution for final packaging.

### 2.4. X-Ray Photoelectron Spectroscopy (XPS) Analysis of the Surface

XPS spectra were measured with a K-Alpha XPS System (Thermo Fisher Scientific, Waltham, MA USA) configured with a monochromated Al Kα (1486.8 eV) 300 W X-ray source, 1.5 mm circular spot size, a flood gun to counter charging effects, and an ultrahigh vacuum (<10^−8^ Torr).

### 2.5. Wettability Assessment by Static Contact Angle

Surface water was removed from the sample using KIMTECH wipe paper (Kimberly-Clark Professional, Roswel, GA, USA), and the sample was then placed on a sample holder. A drop of saline solution (International Standard ISO 18369) [18] of 2 μL was put on the top of the lens. The calculation of the contact angle was performed using FAMAS software (Ver. 3.7.2, Kyowa Interface Science, Saitama, Japan) taking into account the curvature of the sample.

### 2.6. Lipid Adhesion Testing

- Oleic acid adhesion

A solution of Sudan Red in Oleic acid 0.1 *w*/*w*% was prepared at room temperature in a 24-well plate; 1 well was filled with 2 mL of lipid solution. Residual liquid was removed from the surface of the CL with paper tissue. The lens was transferred to the lipid solution for 1 min. It was then dipped into saline solution for rinsing. The contact lens was transferred to a Petri dish filled with ISO saline and a picture was taken using an optical microscope (OLYMPUS, SZX16, Tokyo, Japan).

- Cotton Seed oil adhesion

A solution of Sudan Red in Cotton Seed oil 0.1 *w*/*w*% was prepared at room temperature in a 6-well plate; 1 well was filled with 3 mL of lipid solution. A sponge made of polyvinyl alcohol was soaked in lipid solution for 1 h. Residual liquid was removed from the surface of CL with paper tissue. The soaked sponge was put on the lens, then a metal weight was loaded above it. After 15 s, the lenses were dipped in saline solution for rinsing. The lenses were transferred to a Petri dish filled with ISO saline solution. Pictures were taken using an optical microscope (OLYMPUS, SZX16, Tokyo, Japan).

### 2.7. Cytotoxicity Testing by Colony Formation

Cytotoxicity testing was performed using the colony formation method and direct contact, following the ISO 10993-5 standard, section 8.3 [19]. A known aliquot of continuously stirred cell suspension (V79 Cells; Chinese Hamster, JCRB0603) was used. Initially, positive control and negative control samples were placed at the bottom of the 24-well plate. About 50 cells were seeded into each well (24-well culture plate). After 3 to 5 h of incubation, test samples were placed at the bottom of the well. The samples were incubated for 6 days at 37 °C, with 5% CO_2_ using Minimum Essential Medium (MEM10) with 10% fetal bovine serum (FBS) as the culture medium. The grown colonies were treated with appropriate chemicals/dyes and were counted, and the colony formation rate (CFR) was calculated. The whole series of cell viability tests was conducted over a 14-day period.

### 2.8. Antimicrobial Test

The antimicrobial activity of the coated contact lenses was evaluated according to the Japanese Industrial Standards (JIS) Z 2801 standard [20]. This method involved using both Gram-negative *Escherichia coli* (*E. coli*; IFO3972, Institute for Fermentation, Osaka, Japan) and Gram-positive *Staphylococcus aureus* (*S. aureus*; NBRC13276, National Institute of Technology and Evaluation, Osaka, Japan) for testing microorganisms. Brass was used as the positive control. The procedure included inoculating the test organisms onto the surface of the coated lenses and incubating them under required specified conditions. After the incubation period, the number of viable bacteria on the test samples was compared to that on the control samples. The reduction in bacterial viability was calculated and expressed as the logarithm of the number of viable cells on the negative control divided by the number of viable cells on the test sample.

### 2.9. Contact Lens Properties and Parameters

Contact lens parameters and properties were measured in accordance with the International Standard ISO 18369, parts 3 and 4. This included the evaluation of geometric parameters such as diameter, curvature and central thickness, as well as other critical properties like water content, optical transmittance, UV absorbance and oxygen permeability. Each measurement was performed using standardized equipment and protocols to ensure consistency and reliability. The optical transmittance and UV absorbance were measured with a spectrophotometer, while oxygen permeability was assessed using a polarographic method. Water content was determined gravimetrically, and geometric parameters were measured using specialized instruments designed for contact lens evaluation.

## 3. Results

### 3.1. Technological Process

**Figure 3:**
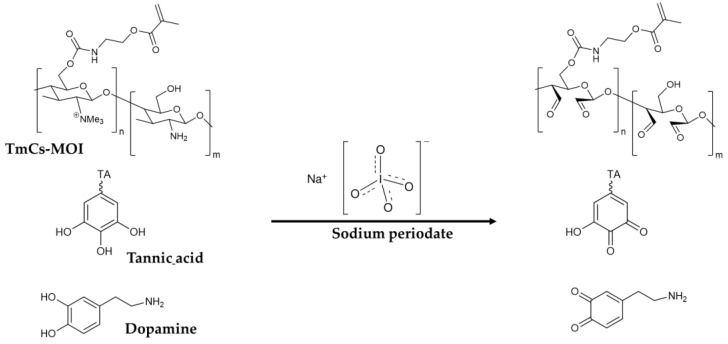
Schematic representation of the coating molecules of dopamine, tannic acid and TmCs-MOI before and after oxidation with sodium periodate.

From a chemical standpoint, the technological process can be divided into three stages: polyethylene imine adsorption; oxidation (Figure 3: Schematic representation of the coating molecules of dopamine, tannic acid, and TmCs-MOI before and after oxidation with sodium periodate); and co-deposition of tannic acid, dopamine and chitosan derivative.

First, bulky molecules of branched high-molecular-weight polyethylene imine are adsorbed on the surface of the contact lens polymer while it is hydrated by an aqueous PEI solution. It should be noted that the contact lens undergoes specific atmospheric plasma surface pretreatment, leading to the creation of various functional groups on the surface such as hydroxyl, carboxyl and amine groups. PEI, a polycationic polymer, possesses a high density of positive charges due to protonated amine groups, which electrostatically interact with negatively charged sites on the contact lens polymer surface. Additionally, the presence of functional groups on both the PEI and polymer surface facilitates hydrogen bonding and other weak interactions, thereby further stabilizing the adsorption. The adsorbed layer of PEI, rich in primary amines, creates a solid basis for further co-deposition of phenolic compounds and chitosan derivatives, as well as forming covalent bonds with them.

Next, a mixture containing tannic acid, dopamine and chitosan derivatives undergoes oxidation by sodium periodate. The phenolic molecules form quinone reactive groups, initiating spontaneous oxy-polymerization [21]. Simultaneously, chitosan oxidation yields aldehyde reactive sites [17], forming covalent bonds via Schiff base mechanisms with the primary amines. This results in all three molecules being deposited onto the surface and bonding with the adsorbed PEI layer. Tannic acid serves as the main coating compound due to its minimal coloring effect, while dopamine is essential for co-deposition, providing a primary amine for Schiff base formation. Finally, oxidized chitosan acts as a macromolecular crosslinker due its dialdehyde reactive sites, enhancing the coating’s hydrophilic properties.

### 3.2. XPS Examination of Surface Modifications

Dried contact lens samples were examined by XPS to analyze the changes in the chemical composition of their surface after treatment. The sample consisted of two lenses: one analyzed from the Front Curve (FC) and the other from the Back Curve (BC).

**Figure 4:**
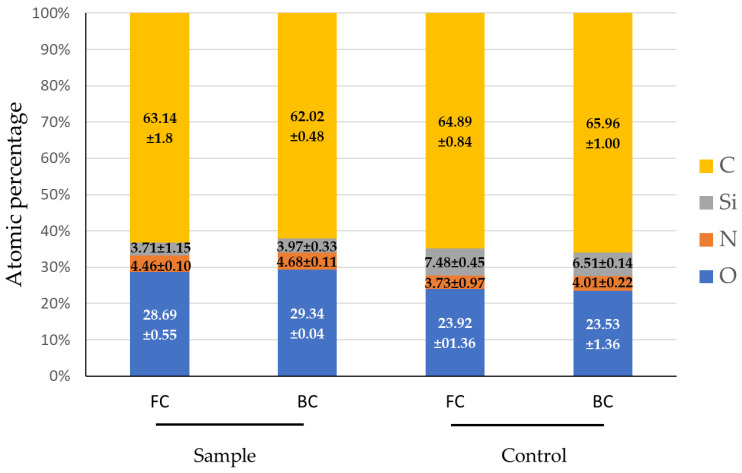
The atomic percentage (average of triplicate) of elements on the modified (sample) and non-modified (control) contact lens surfaces (FC—Front Curve and BC—Back Curve) detected and quantified by XPS.

Atomic percentages were calculated to quantify these changes, as depicted in Figure 4. Four elements were detected and quantified: carbon, silicon, nitrogen and oxygen. There was an increase in the oxygen and nitrogen percentages, which was characteristic of the coating, while silicon, which was the most exposed element on the untreated surface, decreased. These findings suggest that the initial siloxane polymer surface was overlaid with a thin layer of molecules containing oxygen and nitrogen.

Similar trends have been described in the literature. For example, Willis et al. [22] analyzed the surface of phosphorylcholine-coated silicone hydrogel lenses using XPS. Their findings showed that the silicon content decreased by about half, while the oxygen and nitrogen levels increased by 12% and 15%, respectively.

### 3.3. Wettability and Lipid Adhesion

**Figure 5:**
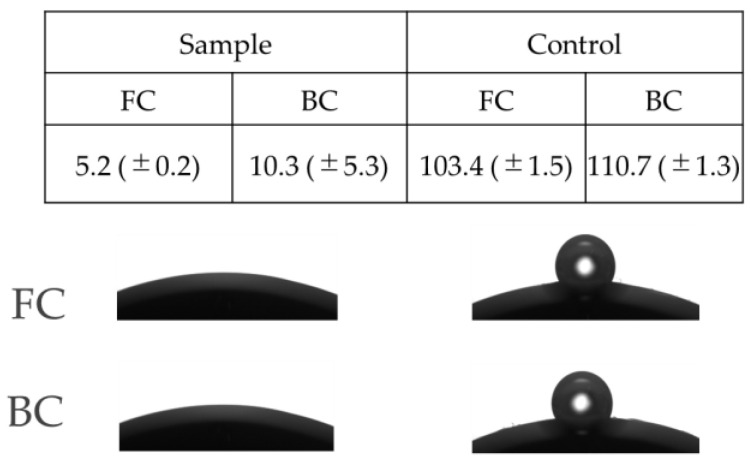
Variation in surface wettability of the modified contact lens (sample) and non-modified one (control). All measurements were performed in triplicate.

Wettability is a critical property for silicone hydrogel contact lenses as it directly impacts the contact lens wearer’s comfort. Good wettability ensures the stability of the tear film, which is crucial for reducing dryness and irritation. Additionally, wettability helps to prevent the accumulation of protein and lipid deposits on the contact lens surface, enhancing the overall ocular health and comfort. A contact angle below 60° is considered optimal to ensure the necessary level of biocompatibility for contact lenses. The samples modified with the polyphenol co-deposition method demonstrated a dramatic improvement in the surface wettability. The contact angle decreased from over 100° to less than 10° degrees (Figure 5).

The slight difference between the results obtained for the Back Curve (BC) and Front Curve (FC) can be attributed to the specific manufacturing process, which results in the unequal wettability of the surface due to variations in the pretreatment process.

**Figure 6:**
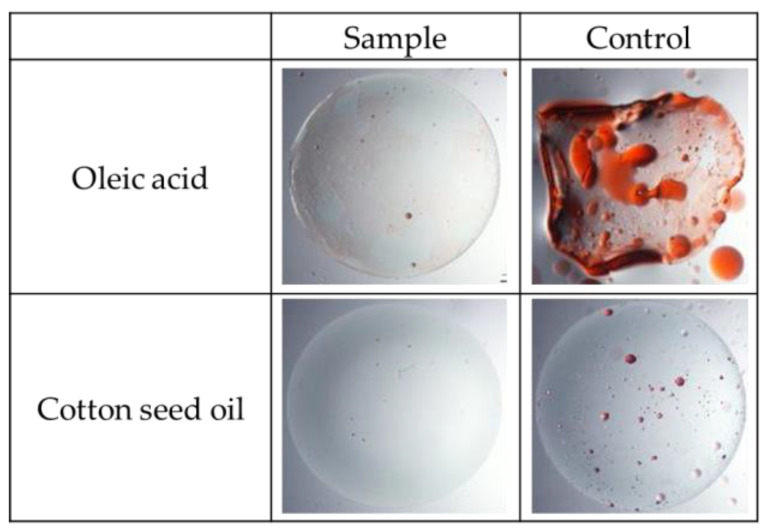
Variation in lipid adhesion of the modified contact lens (sample) and non-modified one (control).

The improvement in the wettability of the contact lenses led to excellent lipid-repellent properties. Two lipid adhesion tests were conducted using Oleic acid and Cotton Seed oil. These tests simulate the potential contamination of the contact lens in the eye during the wearing process with various lipids present in tears. As shown in Figure 6, it was almost impossible to create any kind of lipid adherence to such a highly hydrophilic surface.

### 3.4. Biocompatibility and Antimicrobial Efficacy Tests

**Figure 7:**
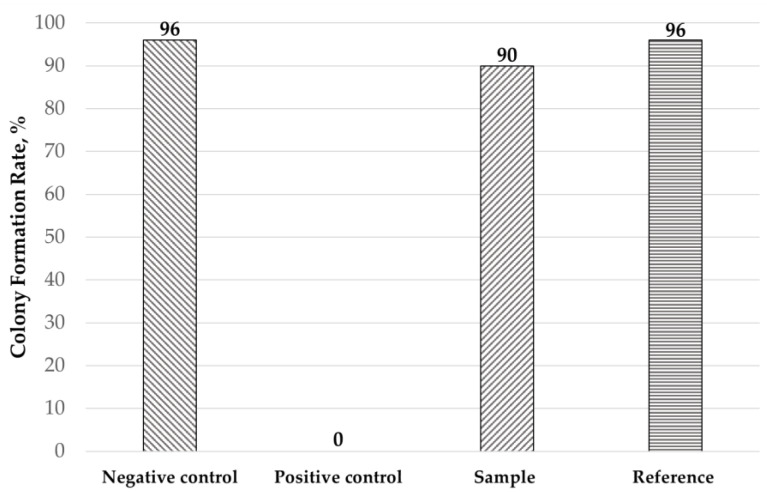
The result of the cytotoxicity test. Cell viability was not affected.

The biocompatibility of the treated contact lenses was confirmed via the colony formation test. As shown in Figure 7, the treated contact lens does not demonstrate any cytotoxicity, as the colony formation rate is over 70%. Therefore, it can be considered safe for further human testing, including a tear film stability test and comfort assessment.

It is well known that both quaternized chitosan [23] and tannic acid [24] may demonstrate antimicrobial activity. The presence and effectiveness of these antimicrobial properties of the coated lenses were assessed following the procedure of the Japanese Industrial Standards (JIS) Z 2801 standard. In this test, both Gram-positive and Gram-negative bacteria were used, with brass serving as the positive control. The reduction in their viability was calculated and expressed as the logarithm of the number of viable cells in the negative control divided by the number of viable cells in the test sample. If the result is over 2.0 for both types of microorganisms, the product may be considered antimicrobial.

A sufficient level of antimicrobial activity was observed against *Staphylococcus aureus*, as shown in Table 1, reaching a value of 2.1. However, only limited activity was detected against the Gram-negative *E. coli*. Therefore, overall, the coated contact lens did not demonstrate enough antimicrobial efficacy to be considered effective. This difference in effectiveness can be attributed to the unique cell wall structure of *E. coli*, which includes an outer membrane that acts as a barrier, reducing the interaction between the positively charged coating compounds and the negatively charged bacterial surface [25]. In contrast, the thicker peptidoglycan layer in Gram-positive bacteria like *Staphylococcus* is more accessible to antimicrobial agents. Additionally, the charge density on the coated surface may not be sufficient to generate strong electrostatic interactions capable of effectively damaging or penetrating bacterial cells, particularly in the case of Gram-negative bacteria like *E. coli*, which have a more complex outer membrane.

### 3.5. Contact Lens Parameters and Properties

To ensure that the coating does not impact the intrinsic properties of the contact lens, a series of evaluations were conducted following the guidelines outlined in the ISO 18369 standard. Initially, geometric parameters such as the diameter, curvature and central thickness were evaluated. Subsequently, properties including the water content, optical characteristics, and oxygen permeability were measured. All of the obtained values were compared to the control, as listed in Table 2.

Minor variations in the key parameters and properties may be observed. Among these, the most notable variations were observed in the light transmittance and UV transmittance. Specifically, the light transmittance decreased by 3 percent, while the UV transmittance showed an average reduction of 2 percent. These changes are likely due to the inherent tinting effect of the phenolic compounds used in the coating process, namely, dopamine and tannic acid. The level of extractables was measured to determine if the coating caused any leaching from the resulting lens. It was observed that the TOC (Total Organic Carbon) levels remained within the specified range, indicating that the coating did not introduce any contaminants into the contact lens. Finally, there was no significant change in terms of the oxygen permeability (Dk), ensuring that the essential properties of the contact lens were maintained.

## 4. Discussion

The technological process was designed with a primary focus on ensuring its suitability for industrial application, taking into account rigorous restrictions and requirements. The specific idea was to apply the technology to the manufacture of daily disposable silicone hydrogel contact lenses. Therefore, the main goals were a short modification time and an unexpensive, easily scalable and technologically simple procedure. To achieve the necessary simplicity of the process, each component was prepared as an aqueous solution. The reaction was then conducted directly in the contact lens blister prior to filling it with a shipping solution and subsequently sterilizing it by autoclaving. The overall process takes less than 15 min.

The resulting lenses demonstrated an extremely hydrophilic surface, with a static contact angle below 10°, surpassing any known contact lenses on the market. Consequently, lipid adhesion, which is crucial for the comfort and safety of any contact lens, was reduced to negligible levels. These results suggest a strong potential for superior physiological performance, leading to enhanced end-of-day comfort levels. Furthermore, the improved wettability is expected to positively contribute to tear film stability. Disruption of the normal tear film often results in an uncomfortable dry sensation, so maintaining the tear film stability is essential for the contact lens wearer’s comfort. In the long term, the systematic disruption of the tear film becomes one of the key factors leading to dry eye syndrome, alongside contributors such as environmental conditions, aging, and underlying health conditions. Therefore, ensuring the adequate wettability of the lens and preventing deposit buildup are important protective measures to reduce the risk of developing dry eye symptoms, including irritation, redness, a gritty sensation, and blurred vision.

The validated cytotoxicity test is the first step for further clinical testing of the modified contact lenses.

In addition, partial antimicrobial activity was demonstrated against Gram-positive Staphylococcus. Given the possibility of post-grafting using the available reactive groups on the surface, we can envision further improvements to the antimicrobial efficacy by incorporating additional antimicrobial agents such as antimicrobial peptoids [26] or zwitter-ion molecules [27]. The interest in antimicrobial properties is minor in the case of daily disposable lenses but becomes significant for extended-wear lenses or rigid gas-permeable lenses. The modification process described herein can be applied to all types of contact lenses with minimal modifications for each specific case.

## 5. Conclusions

This study demonstrated a promising method for the surface modification of hydrophobic contact lens polymers. Through the specific combination of various approaches, we developed an industry-friendly method that is both rapid and technologically simple. This method involves straightforward dip coating in two different aqueous solutions, making it suitable for the high-scale production of daily disposable contact lenses. The resulting lenses were found to be extremely hydrophilic and consequently lipid-repellent. It was shown that the coating did not negatively affect the main intrinsic properties of the contact lens or its cytotoxicity. Additionally, there is strong potential for the further development of broad-activity antimicrobial coatings based on the described methodology. Based on the laboratory results, we expect the superior physiological performance of the resulting product in terms of comfort and tear film stability.

## Figures and Tables

**Table 1:** Result of antimicrobial efficacy test against *Staphylococcus aureus* (*S. aureus*) and *Escherichia coli* (*E. coli*).

*S. aureus*	Activity	*E. coli*	Activity
Sample	2.1	Sample	0.3
Positive control	3.7	Positive control	4.0
Specification	≥2.0	Specification	≥2.0

**Table 2:** Summary of contact lens parameters and property testing.

		Sample	Control
**Parameters**	Diameter (mm)	13.90 (±0.033)	14.03 (±0.106)
	Back Curve (mm)	8.30 (±0.091)	8.29 (±0.059)
	Central Thickness (mm)	0.071	0.072
**Properties**	Water content (%)	55	55
	Luminous transmittance		
	(2°,%)	95	98
	(10°,%)	95	98
	UV transmittance		
	(UV-A,%)	14	16.8
	(UV-B,%)	2.8	4.7
	Refraction	1.404	1.406
	Oxygen permeability Dk	61	64
	Total Organic Carbon (µg/lens)	5.4	4.2

## Data Availability

The original contributions presented in the study are included in the article, further inquiries can be directed to the corresponding author.
